# Physical activity knowledge, attitudes and behaviours of pre-clinical medical students attending an Australian university

**DOI:** 10.1186/s12909-022-03695-y

**Published:** 2022-08-23

**Authors:** Shannon Sahlqvist, Brenton Rees, Samantha Hoffmann, Scott McCoombe, Giuseppe Santoro, Peter Kremer

**Affiliations:** 1 Institute for Physical Activity and Nutrition (IPAN), School of Exercise and Nutrition Sciences, Deakin University, Geelong, 3216 Australia; 2 Centre for Sport Research (CSR), School of Exercise and Nutrition Sciences, Deakin University, Geelong, 3216 Australia; 3 Medical Education Unit, Medical School, The University of Western Australia, Perth, 6009 Australia

**Keywords:** Medical education, Exercise, General practice

## Abstract

**Background:**

Through the provision of advice and counselling, general practitioners (GPs) play an important part in promoting physical activity (PA). Lack of knowledge is a key barrier to engaging in such practice. Little is known about the knowledge and attitudes of current medical students and their preparedness to engage in PA promoting practice in the future. This study aimed to investigate the PA knowledge, attitudes and behaviours of medical students attending an Australian university.

**Methods:**

A sample of 107 pre-clinical medical students from an Australian university completed an online survey. Questions asked about age, sex and past-week PA behaviour (using the International Physical Activity Questionnaire-Short form) as well as understanding of key PA messages and perceptions of the role of a GP, confidence to engage in PA promoting practices and satisfaction with current medical school training (responses were on 5-point Likert scale). Descriptive statistics (proportions, means) were used to summarise demographic and attitudinal measures.

**Results:**

Almost all students (92%) were categorised as being moderately or highly active in the past-week. Knowledge of key PA messages was moderate (3.6 ± 0.9), however understanding of key messages about the dose of PA varied (ranging from 0% to 80.4% agreement). GPs were regarded as having a role to play in promoting PA; with high agreement that discussing the benefits of PA is a part of the role of a GP (4.7 ± 0.5). There was only moderate agreement that participants had received training in the health benefits of PA (3.1 ± 1.0) and in PA counselling (3.2 ± 1.0). Students indicated lower levels of satisfaction with this training (2.5 ± 0.9).

**Conclusions:**

Students in this study were typically physically active, had positive attitudes toward PA and felt that it was the role of the GP to engage in PA promoting practices. Students understood key PA messages, and while they reportedly received some training in providing PA counselling, they were somewhat dissatisfied with this training.

## Introduction

Physical activity (PA) helps to prevent, and treat, a number of chronic diseases including coronary heart disease, type 2 diabetes, breast cancer, colon cancer and dementia, making it one of the ‘best buys’ in public health [1, 2]. In Australia, however, as in much of the Western world [3], rates of PA are low with population estimates indicating that just over half (55%) of adult Australians are participating in sufficient PA [4].

Medical practitioners can play a pivotal role in helping combat population levels of inactivity [5]. Research suggests that general practitioners (GPs), in particular, are perceived as a credible source of information [6–8] and subsequently can be a powerful motivator for behaviour change [8]. They have wide population reach, particularly among those traditionally hard-to-reach [8, 9]. GPs can engage in a range of PA promoting practices from providing written information and resources to advice and counselling and these have been shown to be effective over a 12-month period [10, 11]. Despite this, GPs do not typically discuss PA with their patients during a standard consultation, and when they do, this advice is usually general in nature and brief [9, 12, 13]. Moreover, the advice is typically only offered to those with underlying chronic conditions, highlighting a missed opportunity to provide advice to those who may be inactive but otherwise healthy [14]. This is, in part, because GPs report a lack of both time and knowledge, and feel uncomfortable providing PA advice, particularly if they are inactive themselves [15].

PA is a complex behaviour that requires prescription of an individualised ‘dose’ to be maximally effective and the use of evidence-based strategies to promote adherence. Where GPs lack the knowledge, skills and more pragmatically, time, to provide this advice, one strategy may be to refer patients to an exercise specialist, a practice that has been in place across several countries including the UK, New Zealand and Australia since the 1990s [16]. In Australia, this is an accredited exercise physiologist (AEP), a tertiary qualified allied health professional with the capability to design and deliver safe exercise programs to prevent and manage chronic medical conditions [16, 17]. In 2006, AEPs were formally recognised as allied health professionals under Medicare (the government funded health care system) enabling patients with acute, sub-acute or chronic medical conditions, injuries or disabilities to claim rebates for services provided. Within Australia, GP access to AEPs differs greatly. Some medical centres have AEPs fully integrated into their practice, while for other GPs (i.e., those working in rural and remote areas) there is little opportunity for their patients to access them. Evidence suggests that GP referrals to AEPs is underutilised. In 2012, it was estimated that less than 1% of patients with overweight / obesity or type 2 diabetes were referred to an AEP [18]. Similarly, in 2016, it was estimated that 1.44 per 1000 visits to a GP were referred to an AEP [16].

As future doctors, many medical students will have an important role to play in promoting PA and therefore it is important to understand their preparedness to engage in PA promoting practices. Research in Canada indicates that medical students report higher motivation and lower confidence to engage in PA promoting practices [19]. Moreover, that study, as well as studies in the UK [20] and Canada [21], suggest that knowledge of current PA guidelines is moderate to low with around 50 – 60% of students able to identify the aerobic guidelines for adults. This study aims to build on this work by investigating the perceptions of PA related knowledge and skills in an Australian pre-clinical medical student cohort as well as determine their awareness of AEPs and compare differences in this knowledge and awareness by their personal PA behaviour.

## Methods

### Sample selection and recruitment

A convenience sample drawn from the population of pre-clinical (total: *n* = 286; year 1: *n* = 141; year 2: *n* = 145) medical students at one Australian medical school were invited to complete a brief online survey. A member of the research team (BR) invited students during Public Health Medicine lectures. Additionally, flyers advertising the study were posted around the School of Medicine as well as shared on the medical student association Facebook page. To encourage participation, students who volunteered were offered free personal PA advice and a personalised PA program delivered by an accredited exercise scientist who was a member of the research team (BR).

### Context

The medical degree under investigation is a 4-year postgraduate entry program. Years 1 and 2 are primarily pre-clinical with most learning conducted on campus. In these 2-years, there is an emphasis on medical sciences, public health medicine and ethics, law and professionalism alongside the fundamentals of clinical practice. With regards to training in PA, the majority is taught within public health medicine whereby students learn about the importance of PA for population health and current PA guidelines, hear from GPs about how they incorporate PA into their patient interactions and learn from recent graduates about effective motivational interviewing with respect to PA. A recent curriculum mapping activity indicated that this training accounts for approximately 4 h of all learning activities delivered across both years. By comparison, a recent study found that 4-year programs in Australia deliver an average of 6.6 h of PA training, while 6-year programs deliver an average of 12.3 h [22]. AEPs do not have any direct role within the curriculum and medical students are not directly exposed to AEPs as part of their studies but are provided with information about their role by other members of the teaching team.

### Survey instrument

Standard items assessed age, sex, year of medical school and field of undergraduate study. Using the International Physical Activity Questionnaire – Short Form (IPAQ-S), participants were asked to report the number of days and the average duration per day, spent walking and in moderate- and vigorous-intensity PA in the past week [23]. The IPAQ-S is commonly used to assess population levels of PA and has acceptable reliability and validity [23].

Regarding knowledge and attitudes, one item asked generally about knowledge of the National PA Guidelines while five items explored knowledge of the key PA messages reflected in these guidelines [24]. These were: Taking the stairs and generally being more active is good for health; Half an hour of brisk walking on most days is sufficient for good health; Several short walks of ten minutes on most days is better than one round of golf a week; To be effective, exercise must be done at a vigorous intensity (reverse coded); and Exercise that is good for your health must make you puff and pant (reverse coded). These items have been used to assess health professional understanding of key PA messages [24]. Three items assessed medical school training, specifically, whether participants had received training on the benefits of PA, and on PA counselling, and whether they were happy with this level of training. Two items asked about their level of confidence in providing PA advice in the future [24]. Perceptions of the importance of role-modelling was assessed using two items that examined the extent to which participants felt that a GP’s own health and PA behaviour impacted on their patients’ [25, 26]. Finally, four items assessed awareness of the role of AEP’s as well as three other health professionals (Physiotherapist, Nutritionist & Podiatrist). All items assessing knowledge, attitudes and awareness were answered on a five-point Likert scale with responses ranging from strongly disagree to strongly agree.

### Procedures

Participants completed the survey online using the Qualtrics survey software (Qualtrics, Provo, UT, USA) during September 2017. Ethical approval for the study was obtained from the Deakin University Human Research Advisory Group – Health [HEAG-H 125_2017] and a waiver of written informed consent approved. Consent to participate was implied by willingness to complete the survey.

### Data treatment and analysis

Descriptive statistics were used to summarise key demographic variables. Scores on the IPAQ-S were treated in accordance with published guidelines [21]. Specifically, weekly time spent in vigorous-intensity PA, moderate-intensity PA and walking was determined by multiplying the frequency (days/week) and average duration of each activity. Each activity was then weighted by assigning a Metabolic Equivalent Value (MET) energy expenditure estimate. For walking this was 3.3 METs, for moderate-intensity PA it was 4.0 METS and for vigorous-intensity PA it was 8.0 METs. These values were then summed to provide a measure of total past week PA (MET.min/week) summarised as median and interquartile range values, and subsequently used to classify individuals’ level of PA as high, moderate, or low. The high activity category corresponds to participating in PA above the current World Health Organisation (WHO) guidelines for aerobic activity, whereas the moderate category corresponds to PA equivalent to the guidelines and the low category corresponds to PA below the guidelines [23].

Responses to the items about the current PA guidelines were categorised as ‘correct’ if a participant ‘strongly agreed’ or ‘agreed’ with the statements following the approach used previously [20, 24]. For all other knowledge and attitudinal items responses were converted to a number (strongly disagree = 1, disagree = 2, neutral = 3, agree = 4, strongly agree = 5) and means and SDs computed. Finally, PA categories were cross-tabulated with the outcome variables and additional analyses run (chi-square for categorical outcomes and t tests for continuous outcomes) to test for differences according to PA level. For these analyses, due to the relatively small number of those classified as low PA we combined the low and moderate PA categories.

## Results

Complete responses were obtained from 107 participants (response rate = 37.4%). There was an even split of students in their first (51%) and second year (49%). Just under a third (29%) of students were aged 20 – 22 years, 49% were aged 23 – 25 and 15% were aged 26 – 28, the majority (60%) were female, and most (79%) had completed an undergraduate degree in science. The median total PA was 2267 MET-minweek^−1^ (IQR = 1398, 3680). Assuming that moderate-intensity PA is equivalent to 4 METs, this equals approximately 9 h of moderate-intensity PA per week. Based on weekly PA values, the majority (56%) of participants had a high PA status, while about a third (36%) had a moderate, and a smaller proportion (8%) a low, PA status.

### Knowledge of PA & role of allied health professionals

While self-reported familiarity with the National PA Guidelines was moderate (mean = 3.6) understanding of key aspects of the guidelines varied. Approximately 80% of participants correctly disagreed with the statement that exercise must be done at vigorous intensity to be effective. Likewise, 70% correctly disagreed with the statement that exercise that is good for you must make you puff and pant. In contrast, however, just over 50% of participants agreed that half an hour of brisk walking on most days is sufficient for health. All participants correctly determined that taking the stairs and being generally more active is good for health. Awareness of the role of most allied health professionals was moderately high (i.e., means ≥ 3.9), however, participants were less aware of the role of an AEP relative to the other health professionals (see Table 1).

**Table 1:** Knowledge of PA messaging and role of health professionals

**Knowledge of key PA messages**	**Correct response**^**a**^** n (%)**
Taking the stairs and being generally active is good for health	107 (100)
Half an hour of brisk walking most days is sufficient for good health	62 (57.9)
To be effective, exercise must be done at a vigorous intensity (reversed)	86 (80.4)
Exercise that is good for you must make you puff and pant (reversed)	75 (70.1)
Several short walks of 10 min on most days are better than one round of golf a week	75 (70.1)
**Knowledge of PA guidelines**^**b**^	**mean (SD)**
Knowledge of national PA guidelines	3.6 (0.9)
**Knowledge of health professionals**^**b**^	**mean (SD)**
AEP	3.1 (1.1)
Nutritionist	4.1 (0.6)
Podiatrist	3.9 (0.8)
Physiotherapist	4.3 (0.6)

^a^Agree / Strongly agree = Correct; ^b^Strongly disagree = 1; Disagree = 2; Neutral = 3; Agree = 4; Strongly agree = 5

There was strong agreement (i.e., most means > 4.0) that GPs have a role to play in promoting PA and that the PA behaviour of GPs can positively influence their counselling practice. While participants indicated moderate agreement that they had received training in PA, there was low agreement (mean = 2.5) that their current training was adequate. Students were in some agreement that that they would be confident in providing PA advice to their patients (mean = 3.6) and this was less true for the provision of specific advice (mean = 3.1; see Table 2).

**Table 2:** Perceptions of medical curriculum, future role modelling and the provision of counselling

Item	mean (SD)^a^
**Medical training PA**	
During my lectures/seminars in my current medical degree I have received extensive teaching about the benefits of PA for health	3.6 (1.0)
During my lectures/seminars in my current medical degree I have received teaching on PA counselling	3.2 (1.0)
I am happy with my medical school training in PA counselling (for example, training in talking to patients about PA)	2.5 (0.9)
**Confidence providing PA**	
At this stage of my medical degree, I am confident in counselling patients on PA	2.9 (1.0)
In the future, I will feel confident in giving general advice to my patients on increasing their PA	3.6 (0.9)
In the future, I will feel confident in providing specific advice to my patients about increasing their PA	3.1 (1.0)
**Importance of role modelling PA**	
Good PA habits of the general practitioner can encourage their patients to be active	4.1 (0.8)
To effectively encourage patient adherence to an active lifestyle, a GP must adhere to one himself or herself	3.8 (1.0)
**Role of general practitioners in PA promoting practices**	
Discussing the benefits of PA is a part of the role of a GP	4.8 (0.5)
Suggesting ways for people to increase their PA is an important part of the role of a GP	4.6 (0.6)

^a^Strongly disagree = 1; Disagree = 2; Neutral = 3; Agree = 4; Strongly agree = 5

Knowledge of key PA messages, as well as perceptions of the medical curriculum, future role modelling and PA counselling did not differ according to participants’ level of PA (see Table 3). The only measure indicating a significant group difference was the more general item relating to knowledge of PA guidelines where high PA students indicated higher levels of knowledge than their low/moderate PA peers (Low/Mod PA: mean = 3.2 (1.0); High PA: mean = 4.0 (0.8); *t* = -4.35, *p* < 0.001).

**Table 3:** PA knowledge and perception of medical curriculum, future role modelling and PA counselling for PA groups

	**PA category**		**χ**^**2**^	***df***	***p***
**Variable**	**Low-Mod**	**High**			
**Knowledge of key PA messages**	**Correct, n (%)**	**Correct, n (%)**			
Taking the stairs and being generally active is good for health	47 (100.0)	60 (100.0)	–	–	–
Half an hour of brisk walking most days is sufficient for good health	25 (53.2)	37 (61.7)	.47	1	.49
To be effective, exercise must be done at a vigorous intensity (reversed)	40 (85.1)	46 (76.7)	.72	1	.40
Exercise that is good for you must make you puff and pant (reversed)	37 (78.7)	38 (63.3)	2.29	1	.13
Several short walks of 10 min on most days are better than one round of golf a week	31 (66.0)	44 (73.3)	.38	1	.54
**Knowledge of PA guidelines**	**mean (SD)**	**mean (SD)**	***t***	***df***	***p***
Knowledge of national PA guidelines	3.2 (1.0)	4.0 (0.8)	-4.35	90	< .001
**Medical training PA**					
During my lectures/seminars in my current medical degree I have received extensive teaching about the benefits of PA for health	3.7 (0.9)	3.5 (1.0)	1.19	105	.24
During my lectures/seminars in my current medical degree I have received teaching on PA counselling	3.4 (0.9)	3.1 (1.0)	1.34	105	.18
I am happy with my medical school training in PA counselling (for example, training in talking to patients about PA)	2.6 (0.9)	2.4 (0.9)	.82	105	.41
**Confidence providing PA counselling**					
At this stage of my medical degree, I am confident in counselling patients on PA	2.6 (0.8)	3.0 (1.1)	-1.90	105	.06
In the future, I will feel confident in giving general advice to my patients on increasing their PA	3.6 (0.9)	3.6 (0.9)	-.46	105	.65
In the future, I will feel confident in providing specific advice to my patients about increasing their PA	3.0 (0.9)	3.1 (1.1)	-.70	105	.49
**Importance of role modelling PA**					
Good PA habits of the general practitioner can encourage their patients to be active	3.9 (0.8)	4.2 (0.8)	-1.66	105	.10
In order to effectively encourage patient adherence to an active lifestyle, a GP must adhere to one himself or herself	3.7 (1.0)	3.8 (1.0)	-.70	105	.48
**Role of GPs in PA promoting practice**					
Discussing the benefits of PA is a part of the role of a GP	4.7 (0.5)	4.8 (0.5)	-1.06	105	.29
Suggesting ways for people to increase their PA is an important part of the role of a GP	4.5 (0.6)	4.7 (0.5)	-1.61	105	.11

## Discussion

This study investigated pre-clinical medical students’ knowledge of, and training in, PA, as well as their attitudes towards the role of GPs in providing PA promoting practices. Broadly, our findings mirror those from the UK [20] and Canada [25] which indicate that medical students regard PA as important in their profession yet are dissatisfied with their training. In this study, participants overwhelmingly agreed with statements that providing PA advice and counselling were part of a GPs role yet reported low to moderate satisfaction with the training received. This contrasts with findings from a study of Australian medical school program leaders; noting that they did not observe a strong student interest in receiving training in PA promotion [22].

Intuitively one might expect that the two items which asked about medical training in PA to be answered with less variability (i.e., smaller SDs) than other items as they were asking about ‘actual’ training, however, this was not the case. We compared responses to these two items by year of medical training and found differences in the recorded means but not in variability. In terms of medical training into the benefits of PA, first year students recorded a mean = 3.5 (SD = 1.0) compared with a mean = 3.7 (SD = 0.9) in second year students. Similarly, in terms of medical training in counselling of PA, first year students recorded a mean = 3.0 (SD = 1.1) compared with second year students who recorded a mean = 3.5 (SD = 0.8). It is possible that the variation reflects different perceptions among medical students about what constitutes *adequate* training.

Knowledge of key PA guidelines and key PA messages was moderate. This is somewhat encouraging and indicates that many medical students will disseminate correct information about PA to their future patients, however the findings also indicate that additional training is required to ensure that all students are aware of the minimum dose of PA required for health benefit. Although not directly comparable, these findings nonetheless suggest that medical students in Australia may be better prepared than their counterparts in other countries. For example, 40% of final year UK medical students stated that they were aware of the current guidelines and 60% correctly answered prompted questions about the guidelines [20]. More recently, a survey of final year medical students in the UK categorised 52% as being *unaware* of the current guidelines [27].

Overall, the majority (92%) of participants were categorised as moderately or highly active, participating in PA at, or above, the WHO’s recommendation [28]. This is much higher than national levels, with the most recent data (using the Active Australia Survey) indicating that just over 50% of Australian adults aged 18 – 34 are meeting these recommendations [4]. This difference may be explained, in part, by the measure used. Studies have demonstrated that the IPAQ-S typically overestimates PA participation compared with other self-report measures [29]. It may also reflect the fact that this group of participants is more likely to engage in PA compared with the general population. This is evidenced by a more recent study of Australian university students which showed that approximately 90% of males and 82% of females were meeting PA guidelines in 2018 according to the Active Australia Survey [30]. Proportionately high rates of PA among medical students have also been reported in the US [31, 32] and the UK [25, 27] again, perhaps reflecting, in part, their age and socio-economic postion. Nonetheless it is an encouraging finding as research indicates that GPs who engage in PA are more likely to counsel their patients about PA [33]. Moreover, our own study, and the work of others [19, 33], has shown that both medical students and GPs perceive role modelling to be a precursor to PA promoting practices.

Our findings indicate that medical students report less knowledge of AEPs relative to their knowledge of other health professionals. This is unsurprising, as a previous study of the Australian medical curriculum revealed that only 67% prepared students to utilise referrals to AEPs [22]. The low awareness/ambivalence towards exercise professionals does not appear unique to the medical profession. A recent scoping review found that health care professionals reported being unclear about the role and scope of exercise professionals and lacked knowledge of existing referral practices [34]. Considering our findings that medical students reported low to moderate confidence in providing specific PA advice, and the fact AEPs are an important and cost-effective resource for patients with chronic conditions [35], directly exposing medical students to AEPs (as medical educators for example) and providing additional education into their role and ways to promote collaboration between GPs and AEPs is warranted.

### Strengths and limitations

This study addressed evidence gaps surrounding current medical students’ knowledge of, and attitudes towards, PA promoting practices in the GP setting. Most studies to date have focused on GPs themselves or curriculum content. Gathering the views of medical students is important as they will apply their knowledge in the workforce. Acknowledging that many medical students will not go onto practice as GPs, future studies should explore the views of clinical medical students / registrars.

The study is limited by its use of a survey which is subject to both recall and social-desirability biases. The sample was small and from one Australian university. It may also have had an over-representation of more physically active participants as students already engaged in physical activity and interested in the incentive of PA advice and program may have self-selected to complete the survey, and conversely those not engaged in activity may have chosen not to participate. We cannot say for certain whether participants in our study were representative of medical students attending the University from which they were drawn, or more broadly, students attending other medical schools in Australia, and as such, the generalizability of the study findings is limited. Importantly, whether other Australian medical students would report similar knowledge and awareness of PA promoting practices is unknown. To add to these findings, future work should extend our survey approach taking into consideration the limitations we have highlighted as well as use qualitative methods to allow for a more in-depth exploration of the views of medical students, the current medical curriculum, and the training they receive.

## Conclusions

Most medical students in this study reported participating in moderate to high levels of PA. Broadly, participants felt that promoting PA is central to the role of a GP yet were only moderately confident about providing advice and counselling to their patients. Moreover, while most reported a familiarity with the PA guidelines, some were not aware of key messages about the dose of PA required for health benefit. The disconnect likely reflecting the relatively modest levels of PA teaching within the medical curriculum. Overall, this seemed to engender a degree of dissatisfaction with current levels of PA training. Taken together our findings suggest that medical students are receptive to the inclusion of PA teaching and training within the curriculum. This training should focus specifically on awareness and understanding of PA guidelines as well as the provision of specific advice and counselling strategies. Importantly, this training should include education into the role of exercise professionals, appropriate referral practices and effective interdisciplinary collaboration strategies. Acknowledging the already crammed medical curricula, on-going professional development (i.e., exerciseismedicine.com.au) may hold promise.

## Data Availability

The de-identified dataset used is available from the corresponding author upon reasonable request.

## Abbreviations

AEP: Accredited Exercise Physiologist
GP: General Practitioner
IPAQ-S: The International Physical Activity Questionnaire – Short Form
IQR: Interquartile Range
MET: Metabolic Equivalent
PA: Physical Activity
SD: Standard Deviation
